# New and Emerging Zoonoses^1^

**DOI:** 10.3201/eid1011.040797_05

**Published:** 2004-11

**Authors:** Marguerite Pappaioanou, Thomas Gomez, Cherie Drenzek

**Affiliations:** * Centers for Disease Control and Prevention, Atlanta, Georgia, USA;; † U.S. Department of Agriculture, Riverdale, Maryland, USA;; ‡ Georgia Department of Human Resources, Atlanta, Georgia, USA

**Keywords:** zoonoses, Ebola, monkey pox, *Toxoplasma gondi*, *T. gondii*, influenza A, H7N7

Seventy-five percent of emerging infectious diseases are transmitted from animals to humans. The panel focused on different factors that have caused transmission from animals to humans for four diseases in recent years. The monkeypox infections in humans in the United States in 2003 were the first introduction of the disease into a human population outside Africa. This outbreak resulted from expanded global commerce and travel involving exotic rodents. Humans were infected through contact with ill pet prairie dogs, which had been housed with exotic rodents imported from Africa in April 2003. Laboratory evidence suggested that multiple species of imported rodents were infected, including rope squirrels, Gambian rats, and dormice. Through testing by classic laboratory methods and newer nucleic acid, real-time polymerase chain reaction methods, ultimately 37 human cases were identified. The disease in U.S. patients differed from that previously described in human outbreaks in Zaire/Democratic Republic of Congo. The Centers for Disease Control and Prevention and the U.S. Food and Drug Administration enacted a ban on importation of African rodents and distribution of prairie dogs in the United States to prevent additional introduction of infected animals. Zoonotic concerns remain, including whether monkeypox was transmitted to other North American mammals that may have come into contact with the imported infected rodents. Ongoing studies are focusing on the pathogenesis of infection, incubation period, length of transmissibility, and expression of disease in rodents—all which remain poorly understood.

Several human and animal Ebola virus outbreaks have occurred in West and Central Africa since 2001, causing 313 human cases and 264 deaths. These outbreaks have consisted of multiple simultaneous epidemics caused by different viral strains, with each epidemic resulting from humans' handling of distinct gorilla, chimpanzee, or duiker carcasses. Wildlife die-offs coincided in time and space with human Ebola outbreaks. Based on these results, Ebola virus was proposed as the cause of the rapid local collapse of these wild animal populations. Carcasses were infected by a variety of Ebola virus strains, which suggests that Ebola outbreaks in great apes resulted from multiple virus introductions from an unidentified natural reservoir host. It was proposed that outbreaks in humans could possibly be prevented or predicted by monitoring animal deaths.

The California sea otter, a subspecies named on federal lists of threatened species, is found only along the central coast of California. More than 40% of California sea otter deaths are attributed to infectious agents, including some more typically associated with terrestrial animal and human disease, such as *Toxoplasma gondii*. Brain infection with *T. gondii* has been documented to cause significant numbers of sea otter deaths in California. Growing evidence supports a land-sea connection associated with contamination of the coastal environment, and the source of infection to sea otters.

Domesticated cats, the terrestrial definitive hosts of *T. gondii*, recently have been found to inhabit the coastal California landscape. From 1997 to 2001, *T. gondii* seroprevalence was 42% (49/116), in live sea otters and 62% (66/107) in dead otters. Risk factors positively associated with *T. gondii* seropositivity included male gender; older age; presence in Morro Bay, California; and freshwater outflow exposure. These findings illustrate pathogen pollution in the marine ecosystem and suggest that sea otters could be an indicator species for as-yet-unrecognized human health risks.

An outbreak of highly pathogenic avian influenza A virus subtype H7N7 occurred in the Netherlands beginning in February 2003. The Netherlands Ministry of Agriculture instituted an eradication program to control H7N7 avian influenza in poultry (30 million chickens culled; 255 farms; 20% symptomatic). An unexpectedly high number of H7N7 transmissions occurred in persons directly involved in handling infected poultry; evidence for person-to-person transmission was documented. Enhanced surveillance showed that 453 of an estimated 4,500 people thought to be exposed reported health complaints—349 reported conjunctivitis, and 67 reported other complaints. One veterinarian died. After 19 cases had been identified, all workers received mandatory influenza virus vaccination and prophylactic treatment with oseltamivir. Fifty-six percent of reported H7N7 infections arose before the vaccination and treatment program. By the time full prophylactic measures were reinforced (1 week after the first confirmation of human infection), >1,000 persons from all over the Netherlands and from abroad had been exposed. Poor compliance was observed in the use of personal protective equipment among poultry farmers and cullers.

